# 

**Published:** 1888-05-12

**Authors:** 


PRICE ONE PENNY.
No. 85, Vol. IV.—May 12th, 1888.
[Registered at the General Post Office as a Newspaper.]

Per Annum, 6s. 6d., post free.
Monthly Parts, 6d.; post free, 7½d.
Ditto per Ann., 7s. 6d. post free.

